# Hypercalcemia due to *CYP24A1* variants in five unrelated patients: diagnostic and clinical considerations

**DOI:** 10.1093/jbmrpl/ziaf102

**Published:** 2025-06-13

**Authors:** Jenna L Sarvaideo, Jessica M Colón-Franco, Rajiv Kumar, Joseph L Shaker

**Affiliations:** Division of Endocrinology and Molecular Medicine (Department of Medicine), Medical College of Wisconsin, Milwaukee, WI 53226, United States; Department of Pathology, Cleveland Clinic, Cleveland, OH 44195, United States; Division of Nephrology (Department of Medicine), Mayo Clinic, Rochester, MN 55905, United States; Division of Endocrinology and Molecular Medicine (Department of Medicine), Medical College of Wisconsin, Milwaukee, WI 53226, United States

**Keywords:** Hypercalcemia, Hypercalciuria, Calcitriol, *CYP24A1*, Pregnancy, Lactation

## Abstract

Calcitriol-induced hypercalcemia is most frequently caused by granulomatous and inflammatory conditions such as sarcoidosis as well as lymphoma. Recently, pathogenic *CYP24A1* variants resulting in inability of the 24-hydroxylase enzyme to deactivate 1,25(OH)2D has been found to be a cause of calcitriol-induced hypercalciuria and hypercalcemia in children and adults. Patients may present with hypercalcemia, suppressed PTH, hypercalciuria, and renal stones. We describe 4 young women and 1 man with calcitriol-associated hypercalcemia in whom pathogenic *CYP24A1* variants were found to be the cause. In 2 of the 3 women who became pregnant, hypercalcemia worsened (the calcium was not checked during pregnancy in the third). Lactation was associated with worsened hypercalcemia in the 2 women who breast-fed. In the other woman who did not become pregnant, serum calcium levels varied from high normal to markedly elevated often without an explanation. The male patient was a middle-aged man with a long history of kidney stones and hypercalcemia as well as a family history of kidney stones. Gene sequencing confirmed that each patient had 2 variants in *CYP24A1*. We share 5 cases of a rare condition and further broaden the presentation of *CYP24A1* variants to not only include worsening hypercalcemia in pregnancy, but also during lactation. Further calcium levels may vary markedly in patients with this condition. Physicians should consider pathogenic *CYP24A1* variants in patients with unexplained calcitriol-associated hypercalcemia/hypercalciuria.

## Graphical Abstract

**Graphical Abstract:**
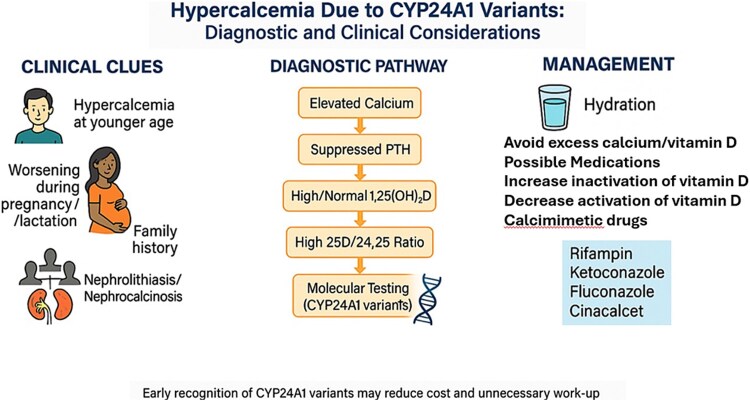

## Introduction

Hypercalcemia in outpatients is usually caused by primary hyperparathyroidism. PTH-independent hypercalcemia is most commonly caused by malignancy or unregulated, extra-renal production of 1,25-dihydroxycholecalciferol (1,25(OH)_2_D or calcitriol) as occurs in granulomatous and inflammatory conditions.^1^

In 2011, Schlingmann et al.^2^ reported infantile hypercalcemia associated with elevated 1,25(OH)_2_D due to pathogenic loss of function variants in the 24-hydroxylase enzyme (*CYP24A1*) which deactivates 1,25(OH)_2_D, the active form of vitamin D. This enzyme converts 1,25(OH)_2_D and also 25(OH)D into their inactive 24-hydroxylated products, 1,24,25(OH)_3_D and 24,25[OH]_2_D, respectively. There have been subsequent reports of adult hypercalcemia, hypercalciuria, nephrocalcinosis, and kidney stones associated with pathogenic variants of *CYP24A1.*^3–5^

Further, worsening hypercalcemia was reported during pregnancy by Shah et al.^6^ and is reviewed by Pilz et al.^7^ and may result in pancreatitis^8^ and be associated with hypertension.^7^ Herein, we describe the cases of 4 women and 1 man with this condition. We confirm the prior reports of worsening of hypercalcemia during pregnancy. We also extend the finding of worsening hypercalcemia to lactation. In the one woman who did not have a pregnancy, very significant fluctuations in calcium level from high normal to marked hypercalcemia occurred without explanation.

## Materials and methods

This is a retrospective chart review approved by the Medical College of Wisconsin (MCW) Institutional Review Board (IRB). Serum/plasma and urine chemistries were measured by routine methods in the respective clinical laboratories. The reference intervals for calcium were 8.4-10.2 mg/dL and phosphorus 2.5-4.8 mg/dL. Intact PTH (normal, 15-72 pg/mL) was measured by electrochemiluminescence immunoassay (Roche Diagnostics). The PTH-related protein (PTHrP) was measured by Labcorp (Esoterix, Endocrine Sciences) (patients 1, 2, and 3), and electrochemiluminescense (Meso Scale Discovery) (patients 4 and 5) and with reference intervals of 0.00-1.99 and <4.2 pmol/L, respectively. 25(OH)D (optimal, 30-50 ng/mL) was measured using the DiaSorin Liaison Assay (DiaSorin). The 1,25(OH)_2_D (normal, 18-78 pg/mL) was measured by liquid chromatography–tandem mass spectrometry (LC–MS/MS) (ARUP Laboratories). The serum 24,25(OH)2D was measured by competitive binding assay (patient 1), HPLC–MS/MS-Labcorp (patient 2), immunoaffinity LC–MS/MS, University of Washington (patient 3), and by LC–MS/MS, Mayo Clinical Lab (patients 4 and 5). Case-specific laboratory tests used in the evaluation of these patients are described below, as applicable.

## Results

### Case 1

A 27-yr-old G2P1 woman presented at about 12 wk of gestation with headache, constipation, nausea, vomiting, and increased thirst. She was found to have severe hypercalcemia (14.9 mg/dL). Her medical history was pertinent for kidney stones and preeclampsia with her first pregnancy, and PTH-independent hypercalcemia (13.8 mg/dL) approximately 1 mo postpartum while lactating 7 yr earlier. Between pregnancies, she had normal serum calcium levels. She denied relevant family history. She was not taking calcium or vitamin D supplements. Her height was 169 cm (66.5″). There were no significant findings on physical exam. Representative biochemical studies are shown in the table and included albumin-adjusted calcium 12.4 mg/dL, ionized calcium 1.72 mmol/L, PTH 7 pg/mL, 25(OH)D 65 ng/mL, PTHrP undetectable, and 1,25(OH)_2_D 163 pg/mL. The 24-h urinary calcium was 528 mg/d The angiotensin converting enzyme activity was normal. The patient had a normal chest X-ray and chest, abdomen, and pelvis MRI. Because of the possibility of an underlying granulomatous process, she was given a trial of prednisone without biochemical response. Biochemical evaluation of her family revealed normocalcemia in her parents and 2 sisters. Her brother had mild hypercalcemia (10.8 mg/dL) with a suppressed PTH. The possibility of *CYP24A1* pathogenic variant was considered. The 24,25[OH]_2_D was 0.41 ng/mL (reference interval 1.2-2.6) (competitive binding assay). DNA sequencing was performed by Molecular Diagnostic Laboratory, and the patient was found to have pathogenic variants in *CYP24A1*. More specifically, she was found to have an in-frame deletion in exon 2 resulting in a deletion of glutamic acid at position 143(c.428_430delAAG; p.Glu143del) and a nonsense variant in exon 8, which substitutes a stop codon for the normal glutamine at position 346 (c.1039C>T; p.Gln346*). She was treated with fluids as well as calcium and vitamin D restriction. Significant hypercalcemia persisted throughout pregnancy and during lactation (as high as 13 mg/dL). When breastfeeding was stopped about 2 mo postpartum, the calcium promptly decreased to normal (9.5 mg/dL, 4 d after stopping nursing) and remained normal (9.5-9.8 mg/dL for the following 3 yr). The PTH remained low/low-normal and the hypercalciuria persisted. Her infant son did not experience hypocalcemia, which would be expected, if his parathyroid glands were suppressed, but rather he had mild hypercalcemia which resolved by 2.5 yr of age. This suggests that he is heterozygous for a pathogenic *CYP24A1* variant. He was clinically normal with normal growth and milestones.

### Case 2

A 24-yr-old woman was seen for hypercalcemia (11.9 mg/dL) with mildly elevated creatinine (1.29 mg/dL). Serum calcium levels from 2-yr earlier were normal. Her medications included vitamin B12, fish oil, vitamin D 2000 IU daily, an oral contraceptive, and low-dose aspirin. The family history was non-contributory, and there was no known consanguinity. Subsequent evaluation of family members revealed normocalcemia in her mother and sister. The calcium levels of her father and 2 brothers are unknown. Her height was 162 cm (63.7″). Representative biochemical studies are shown in the table. The ionized calcium was 1.55 mmol/L with suppressed intact PTH levels of 7 and 6 pg/mL. The 25(OH)D was 57 ng/mL and 1,25(OH)_2_D was high normal/elevated at 74 and 79 pg/mL. The PTHrP was undetectable. The urinary calcium was elevated at 431 mg/24 h. The angiotensin converting enzyme, ANCA, SPEP, and UPEP were normal as was a chest X-ray and CT chest, abdomen, and pelvis. There was no biochemical response to prednisone. The concentration of 24,25[OH]_2_D was undetectable (reference interval 1.8-9.1) (HPLC-MS/MS-Labcorp). *CYP24A1* DNA sequencing was completed by Molecular Diagnostic Laboratory, which revealed a pathogenic variant in *CYP24A1* [homozygous substitution within exon 9 (c.1186C>T; pArg396Trp)]. Her clinical course revealed calcium levels which ranged from 9.8 to 13.0 mg/dL. At times of marked hypercalcemia, creatinine also increased to ~1.5 mg/dL. On one occasion, she had an albumin-adjusted calcium of 12.8 mg/dL shortly after UVB exposure consistent with the known vitamin D sensitivity of this condition. She has been treated with high fluid intake, calcium and vitamin D restriction, avoidance of sun, and use of sunscreen.

### Case 3

A 43-yr-old woman was seen for non-PTH mediated hypercalcemia. The patient delivered a healthy baby boy 2 wk prior to evaluation. Hypercalcemia was incidentally noted on peri-partum labs. She denied symptoms of hypercalcemia. Her height was 155 cm (61.0″). Representative biochemical studies are shown in the table. The serum albumin-adjusted calcium was 13 mg/dL, and ionized calcium 1.73 mmol/L. The 25(OH)D was 60 ng/mL and phosphorus 3.4 mg/dL (see Table 1). Review of old labs revealed unadjusted calcium levels that ranged from 9.6 to 10.7 mg/dL prior to pregnancy. She had 1 episode of renal stones when she was 23-yr-old. She was taking a prenatal vitamin and denied a family history of calcium or parathyroid disorders. The chest X-ray showed no radiographic evidence of cardiopulmonary disease, and an abdominal radiograph showed no abnormal calcifications. Repeat laboratory evaluation 3 wk postpartum revealed albumin-adjusted calcium 10.2 mg/dL and PTH 3.8 pg/mL. The TSH, vitamin A, UPEP, and SPEP were normal. Her 24-h urine calcium was 421 mg/24 h. 1,25(OH)_2_D levels were 78.7 and 77.3 pg/mL. The PTHrP was undetectable. The 24,25(OH)D was low at 0.1 ng/mL (immunoaffinity LC–MS/MS, University of Washington). *CYP24A1* sequencing and deletion/duplication analysis was done at Molecular Diagnostic Laboratory. She was found to be homozygous for a pathogenic variant. The variant detected was (c.1186C>T; pArg396Trp) in exon 9. The patient was advised to stay hydrated and to avoid calcium, vitamin D, and sun exposure. While breastfeeding, her unadjusted calcium ranged from 10.3 to 11.4 mg/dL.

**Table 1:** Summary of biochemistry in the 5 patients.

	**Case, age in years and sex**				
	**Case 1, 27 F**	**Case 2, 24 F**	**Case 3, 43 F**	**Case 4, 55 M**	**Case 5, 35 F**
**Calcium (8.4-10.2 mg/dL)**	12.1	12.3	12.0	11.3	11.0
**Albumin (3.5-5.2 g/dL)**	3.6	4.6	2.8	4.6	3.8
**Albumin-adjusted calcium (mg/dL)**	12.4	11.8	13	10.8	11.2
**Ionized calcium (1.15-1.35 mmol/L)**	1.72	1.53	1.73	1.45	1.44
**Intact PTH (15-72 pg/mL)**	7.0	7.2	3.8	24	3.0
**25(OH) vitamin D (30-100 ng/mL)**	65	57	60	31	61
**Phosphorus (2.5-4.8 mg/dL)**	2.7	4.1	3.4	3.0	4.3
**Creatinine (0.50-1.10 mg/dL)**	1.22	1.40	0.98	1.04	0.50
**Alkaline phosphatase (35-104 U/L)**	55	39	68	80	60
**1,25(OH)_2_ vitamin D (18-78 pg/mL)**	163	79	79	226	147
**24,25[OH]_2_ vitamin D**	0.41 ng/mL (normal, 1.2-2.6)	Undetectable	0.1 ng/mL	<0.10 ng/mL	0.33 ng/mL
**25(OH) vitamin D/24,25[OH]_2_ vitamin D**	158	∞	600	∞	182
**Urine calcium (100-300 mg/24 hr)**	523	431	421	790	90
*CYP24A1*	2 pathogenic variants	Homozygous pathogenic variants	Homozygous pathogenic variant	1 pathogenic variant 1 variant of unknown significance	2 variants of uncertain significance
**Additional information**	No response to prednisone. Marked hypercalcemia during pregnancy and lactation. Normal serum calcium with low PTH and hypercalciuria after lactation	Marked variability in serum calcium. Creatinine increased with worsening hypercalcemia	Hypercalcemia during lactation. Normal to mildly elevated serum calcium before pregnancy	Multiple stones PTH not completely suppressed ***ALPL:*** 1 pathogenic variant Family history of kidney stones (son, brother, father and grandfather)	Normal calcium 4 years before pregnancy. Family history of kidney stones in father. Calcium 9.0 mg/dL 17 days post-partum (not nursing)

*25(OH)D/24,25(OH)_2_D ratio > 80 suggestive of biallelic pathogenic variants of *CYP24A1.*

### Case 4

A 55-yr-old man was seen because of multiple kidney stones (calcium oxalate) dating to his 20s. He also had a long history of hypercalcemia. Review of records revealed hypercalcemia since age 30 (no earlier levels available). There was a family history of kidney stones in his son, brother, father, and paternal grandfather. He was not taking calcium or vitamin D supplements. His height was 178 cm (70.1″). Representative biochemical studies are shown in the table. He had hypercalcemia with markedly elevated urinary calcium and 1,25(OH)_2_D. Interestingly, the PTH was not completely suppressed and ranged from 16 to 38 pg/mL. The PTHrP was undetectable. The 24,25(OH)_2_ vitamin D level was undetectable. He underwent genetic testing through Invitae. He was found to have 1 pathogenic variant of *CYP24A1* (c.443T>C; p.Leu148Pro) and a variant of unknown significance of *CYP24A1* (c.544-17G>A [Intronic]). He also had a pathogenic variant of *ALPL*, but had no clinical evidence of hypophosphatasia and had a normal alkaline phosphatase level (Table 1) and is presumably a carrier. The vitamin B6 level was not measured.

### Case 5

A 35-yr-old woman was evaluated for hypercalcemia (10.8-11.1 mg/dL) at 14 wk gestation (embryo transfer—from her and her partner). She had 2 prior miscarriages and 1 failed prior embryo transfer. There was no prior known history of hypercalcemia and a serum calcium of 4 yr earlier was 9.5 mg/dL. Her dietary calcium was <500 mg daily and she was not taking calcium or vitamin D supplements. There was no history of lithium or thiazide use. She was experiencing fatigue, nausea, vomiting, and constipation. There was no personal history of kidney stones; however, there was a history of kidney stones in her father. Her height was 153 cm (60.2″). Representative biochemical studies are shown in the table. The 1,25(OH)2D was elevated, PTHrP 0.5 pmol/L (normal, <4.2), and PTH suppressed. The ratio of 25OHD to 24,25(OH)_2_ vitamin D was markedly elevated. Genetic testing (Invitae) revealed 2 variants of unknown significance in *CYP24A1* (c.1490A>C; p.His497Pr) and (c.544-17G>A [Intronic])*.* She was treated with self-hydration and was able to maintain albumin-corrected calcium levels of 9.7-10.4 mg/dL. After 17 d of delivery, the albumin-adjusted calcium was 8.9 mg/dL. She was not nursing.

### Summary of cases

We report 5 patients with hypercalcemia in whom pathogenic or likely pathogenic variants of *CYP24A1* appear to be the cause. Hypercalcemia was worsened by pregnancy in 2 patients (no calcium level during pregnancy in the third) and was worsened by lactation in 2 patients. Hypercalcemia was markedly labile in another patient. Patient 4 had 1 pathogenic variant of *CYP24A1* and 1 variant of unknown significance of *CYP24A1*. We do not know if the pathogenic variant caused dominant disease or if the variant of unknown significance is also pathogenic. We cannot explain why the PTH was not completely suppressed in patient 4. In the report of 9 patients with 24-hydroxylase deficiency described by Azer et al.,^9^ one patient had normal PTH levels. Interestingly, there is a report of a patient with *CYP24A1-*associated hypercalcemia and normal (non-suppressed PTH), who had an enlarged parathyroid gland removed with persistent hypercalcemia and then suppression of PTH.^10^ Finally, we do not know if the pathogenic *ALPL* variation in patient 4 could have modified his clinical presentation as hypophosphatasia may be associated with hypercalcemia and hypercalciuria.^11^ However, this is not typically seen in carriers or adult-onset disease. The patient did not have clinical evidence of hypophosphatasia and the serum total alkaline phosphatase was normal. Patient 5 had 2 variants of unknown significance of *CYP24A1*. The presence of calcitriol-associated hypercalcemia with a very high ratio of 25OHD to 24,25(OH)_2_ vitamin D strongly suggests that at least 1 of the variants is pathogenic. We do not have an explanation for the low urinary calcium in patient 5 other than a low calcium intake. Patient 5 had experienced 2 prior miscarriages. We don’t know the causes of her miscarriages and can only speculate on the role of hypercalcemia.

## Discussion

Calcitriol-mediated hypercalcemia is usually caused by non-regulated, extra-renal production of 1,25(OH)_2_D by macrophages in granulomatous and inflammatory conditions, such as sarcoidosis and tuberculosis.^1^ There are several other conditions which cause hypercalcemia by this mechanism (including lymphoma and other malignancies).^1^

Vitamin D metabolism is illustrated in the figure and is reviewed by Bikle.^12^ Vitamin D (produced in the skin or consumed) undergoes hydroxylation in the liver, resulting in 25(OH)D. This is mediated by *CYP2R1* and to a lesser extent *CYP27A1*. The enzyme 1 alpha-hydroxylase (*CYP27B1*) converts 25(OH)D to its active form, 1,25(OH)_2_D, in the proximal convoluted tubule of the kidney. Importantly, 1,25(OH)_2_D is catabolized by *CYP24A1* through a complex process beginning with 24-hydroxylation by 24-hydroxylase. This enzyme also converts 25(OH)D to 24,25(OH)_2_ vitamin D. Another enzyme, *CYP3A4*, which is expressed the liver and intestine, also catabolizes 25(OH)D and 1,25(OH)_2_D.^13^

In recent years, idiopathic infantile hypercalcemia has been found to be associated with bi-allelic pathogenic variants of *CYP24A1*^2^ as well as bi-allelic pathogenic variants of *SLC34A1*.^14^ The latter encodes the sodium-phosphate cotransporter 2A. Loss of function variants of *SLC34A1* result in decreased phosphate reabsorption in the renal tubule with resulting hypophosphatemia which decreases FGF23. The latter results in greater production of 1,25(OH)_2_D and hypercalcemia. Patients with loss of function variants in *CYP24A1* (decreased activity of the 24-hydroxylase enzyme) may have hypercalcemia, hypercalciuria, elevated 1,25(OH)_2_D, and suppressed PTH.^2–5^ Blau syndrome, an autosomal dominant granulomatous condition, caused by pathogenic variants in the *NOD2* gene may cause calcitriol-associated hypercalcemia in children.^15^

Clinical clues to the diagnosis of pathogenic variants of *CYP24A1* in the setting of calcitriol-induced hypercalcemia are young age, family history, and nephrocalcinosis.^9^ This diagnosis is suggested by the finding of low 24,25(OH)_2_D, although 24,25(OH)_2_D may be detectable.^16^ A high 25OHD/24,25(OH)_2_D ratio is strongly suggestive of this diagnosis,^17^ however, definitive diagnosis is best confirmed by molecular testing. Furthermore, heterozygotes that are hypercalcemic or eucalcemic typically have a normal 25OHD/24,25(OH)_2_D ratio.^17^ Another clue to this diagnosis is a high-normal 25(OH)D level in the presence of calcitriol-associated hypercalcemia as was seen in 4 of our 5 patients. Azer et al.^9^ found 25(OH)D levels to be higher in patients with pathogenic *CYP24A1* variants than in sarcoidosis or lymphoma. This is not surprising as patients with the latter conditions have 1,25(OH)_2_D-mediated induction of *CYP24A1*, either directly or via increased FGF23 secretion.^18^ Most patients with hypercalcemia due to pathogenic variants of *CYP24A1* have biallelic mutations; however, heterozygous variants may cause hypercalciuria and kidney stones and sometimes hypercalcemia.^19–21^ Brancatella et al.^20^ reported a family in whom heterozygotes had a biochemical phenotype intermediate between the homozygous proband and wild-type. Additionally, any process that increases calcium or vitamin D may unmask underlying pathogenic variants. The prevalence of *CYP24A1* variants in the general population is unknown; however, Molin et al. found that 35% of a population of patients with hypercalcemia and low PTH (*N* = 72) harbored *CYP24A1* variations with 28% having biallelic variations.^17^

The management of hypercalcemia due to *CYP24A1* pathogenic variants includes calcium and vitamin D restriction as well as hydration. Of note is that calcium and vitamin D restriction have not been shown to be effective and chronic calcium restriction could actually increase oxalate absorption in the gut and increase stone disease. The antifungal drug ketoconazole that decreases 1-hydroxylation of 25(OH)D has been successfully used,^21^ but risk for hepatotoxicity makes this drug less than optimal. Further, it should not be used during pregnancy or lactation. Sayers et al. reported successful treatment of hypercalcemia associated with *CYP24A1* variants with fluconazole.^22^ This drug has a lower risk of hepatotoxicity than ketoconazole. Other risks of imidazoles also include suppression of cortisol and testosterone production.^23^^,^^24^

**Figure 1:**
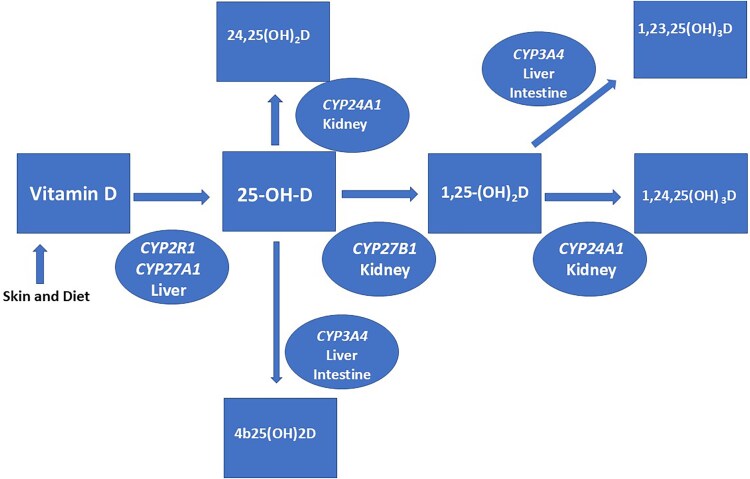
Metabolism of vitamin D.

Rifampin has been successfully used in patients who lack *CYP24A1* function.^25^^,^^26^ Rifampin induces *CYP3A4*^25^^,^^26^ a hepatic/intestinal enzyme which metabolizes vitamin D (see Figure 1). Rifampin has less hepatic toxicity than ketoconazole or fluconazole, and there is an ongoing clinical trial using rifampin in these patients.^27^ A study in a child with idiopathic infantile hypercalcemia due to pathogenic variants of *CYP24A1* revealed a reduction in 1,25(OH)_2_D with short-term treatment with rifampin; however, hypercalcemia did not improve.^28^ However, there is a report of successful rifampin treatment of hypercalcemia due to pathogenic *CYP24A1* variants in a 10-mo-old child.^29^ There was also a recent report of successful treatment of 1,25(OH)_2_D-mediated hypercalcemia due to pathogenic *CYP24A1* variants with cinacalcet.^30^ Additionally, successful treatment of severe hypercalcemia of infancy due to *CYP24A1* variants with pamidronate has been reported.^31^ Bisphosphonates, denosumab, and calcitonin have been used as adjunctive management of postpartum mothers and calcitonin has been used during pregnancy in patients with hypercalcemia due to this condition.^8^

In our report, we describe significant worsening of hypercalcemia during pregnancy, as previously described.^6–8^ This may in part be related to placental production of 1,25(OH)_2_D in the setting of decreased ability to deactivate 1,25(OH)_2_D as both *CYP27B1* and *CYP24A1* are expressed in placental tissue.^32^ However, human and animal data suggest that the maternal kidneys are largely responsible for the marked increase in maternal calcitriol during pregnancy.^33^ Furthermore, we found that hypercalcemia may be worsened during lactation and improve when lactation is stopped. This was not noted in most of the prior cases but may have occurred in the patient reported by McBride et al.^34^ It is possible breastfeeding was not initiated in some of the reported women after delivery because of concern lactation might worsen hypercalcemia. Worsening hypercalcemia during lactation in these patients could be related to intermittent production of PTHrP by the breast as PTHrP is found in high concentrations in breast milk.^35^ We did not find elevated PTHrP during lactation in our patients, but PTHrP secretion in the setting of lactation may be intermittent.^35^ PTHrP-related hypercalcemia has been reported in mammary hyperplasia.^36^ Of note, PTHrP is a less potent stimulator of *CYP27B1* than PTH.^37^ Although controversial, it is possible that intermittent increases in prolactin during lactation may contribute to hypercalcemia by stimulating *CYP27B1*, which drives 25(OH)D to 1,25(OH)_2_D.^38^ Further, it should not be surprising that hypercalcemia can worsen during lactation in patients with *CYP24A1* variants. Women with hypoparathyroidism often require no calcium and vitamin D during lactation.^38^

Another interesting clinical observation is dramatic variability in serum calcium seen in patient 2 without obvious explanation. She also experienced severe hypercalcemia after UVB exposure. This is consistent with hypersensitivity to vitamin D and sun.^39^ Interestingly, discordant clinical courses have been described in brothers with pathogenic variants of *CYP24A1.*^40^

In conclusion, the diagnosis of *CYP24A1* variants requires a high index of suspicion in patients with unexplained calcitriol-induced hypercalcemia. This diagnosis is suggested by the finding of low 24,25(OH)_2_D, although 24,25(OH)_2_D may be detectable^16^ as well as a high 25OHD/24,25(OH)_2_D ratio.^17^ We propose measurement of 24,25(OH)vitamin D and calculation of the 25(OH)D to 24,25(OH)_2_D ratio (25D/24,25D) in patients with unexplained calcitriol-associated hypercalcemia as an early diagnostic test. It is worth noting that this ratio is elevated in patients with biallelic pathogenic variants but is typically normal in heterozygotes.^14^ Hypercalcemia at younger age, positive family history, and nephrocalcinosis are clues to this diagnosis^9^ as is hypercalcemia occurring during pregnancy and lactation. High-normal 25(OH)D levels are also clues to this diagnosis. Although long-term hypercalcemia is also a clue to this diagnosis, the lack of long-term hypercalcemia does not exclude this diagnosis as evidenced by the marked variability in calcium sometimes seen. We believe 25D/24,25D should be done before unnecessary imaging and steroid trials as were done in some of our patients prior to correct diagnosis. Molecular testing is strongly advised in making a definitive diagnosis and should be done in patients suspected of being heterozygotes even if 25D/24,25D is normal. Further, because heterozygous individuals may manifest hypercalciuria and hypercalcemia, genetic and biochemical testing of offspring of affected parents, especially those who carry biallelic *CYP24A1* variants is advised. The degree of hypercalcemia may vary widely and be worsened by pregnancy as well as lactation as well as processes that increase calcium or vitamin D. Management typically includes hydration and vitamin D and calcium restriction, however, these interventions have not been shown to be effective. Drugs that decrease 1-hydroxylation of 25(OH)D or induce enzymes, which inactivate vitamin D may be useful adjuncts and cinacalcet may be effective.

## Data Availability

Data are available upon request.
